# No difference in flexion power despite iliopsoas fatty degeneration in healed hip fractures with large lesser trochanter displacement

**DOI:** 10.1007/s00590-018-2200-4

**Published:** 2018-04-13

**Authors:** Matthias Schenkel, Malwina Kaniewska, Tobias Bühler, Suzanne Anderson, Karim Eid

**Affiliations:** 1 Department of Orthopedic Surgery, Baden State (Cantonal) Hospital, Im Ergel 4, 5404 Baden, Switzerland; 2 Department of Radiology, Baden State (Cantonal) Hospital, Im Ergel 4, 5404 Baden, Switzerland

**Keywords:** Fatty infiltration, Iliopsoas muscle, Proximal femoral fracture, Lesser trochanter, Flexion strength

## Abstract

**Objective:**

To evaluate iliopsoas atrophy and loss of function after displaced lesser trochanter fracture of the hip.

**Design:**

Cohort study.

**Setting:**

District hospital.

**Patients:**

Twenty consecutive patients with pertrochanteric fracture and displacement of the lesser trochanter of > 20 mm.

**Intervention:**

Fracture fixation with either an intramedullary nail or a plate.

**Outcome measurements:**

Clinical scores (Harris hip, WOMAC), hip flexion strength measurements, and magnetic resonance imaging findings.

**Results:**

Compared with the contralateral non-operated side, the affected side showed no difference in hip flexion force in the supine upright neutral position and at 30° of flexion (205.4 N vs 221.7 N and 178.9 N vs. 192.1 N at 0° and 30° flexion, respectively). However, the affected side showed a significantly greater degree of fatty infiltration compared with the contralateral side (global fatty degeneration index 1.085 vs 0.784), predominantly within the psoas and iliacus muscles.

**Conclusion:**

Severe displacement of the lesser trochanter (> 20 mm) in pertrochanteric fractures did not reduce hip flexion strength compared with the contralateral side. Displacement of the lesser trochanter in such cases can lead to fatty infiltration of the iliopsoas muscle unit. The amount of displacement of the lesser trochanter did not affect the degree of fatty infiltration.

**Level of evidence:**

II.

## Introduction

Pertrochanteric fractures (AO/OTA type 31-A1-3) account for one of the most frequent skeletal injuries in patients above 60 years [1]. Treatment of these fractures is usually performed by closed reduction of the fragments and fixation either with an intramedullary nail or a plate. Both lead to a temporary support against the compression and shear forces, thus allowing for early mobilization with at least partial weight bearing.

The pertrochanteric fracture pattern may involve four fragments: the femoral neck, the greater trochanter, the lesser trochanter, and the distal femoral fragment. The lesser trochanter is fractured in half of the patients with proximal femoral fractures [2]. It is widely accepted that the fracture reduction of the two main fragments and the greater trochanter must be as anatomical as possible; however, the lesser trochanter fragment is usually not addressed in the operative procedure at all.

The lesser trochanter represents the insertion site for the iliopsoas muscle, which is one of the largest muscles of the human body.

The iliopsoas is mainly responsible for hip flexion, while the rectus muscle exerts more strength in extension [3–8].

There is only little information on the effect of displacement of the lesser trochanter on hip function, particularly regarding the hip flexion force [9, 10]. This might be because lesser trochanter fracture mainly affects the older population, and patients’ functional demands may be limited to household activities.

In shoulder surgery, it is well recognized that rotator cuff tears with substantial retraction of the tendon relatively quickly lead to fatty infiltration and functional impairment of the affected muscles. The radiological degree of fatty infiltration was first described on computed tomography and has since been adapted for magnetic resonance imaging [11]. In addition, Goutallier et al. [12] introduced the term “global fatty infiltration” as a description of the degeneration of all rotator cuff muscles, which is a prognostic factor for the functional outcome after rotator cuff surgery. It is conceivable that the torn iliopsoas musculotendinous unit may undergo a similar process of fatty infiltration. To our knowledge, there is currently no information in the current literature on the integrity of the psoas and iliacus muscles after substantial retraction of their insertions posttraumatically. We hypothesized that substantial (> 20 mm) fracture displacement of the lesser trochanter would lead to fatty infiltration of the iliopsoas muscle unit and that this would result in impaired flexion force near full extension.

## Methods

### Patients

The present study was approved by the Ethical Committee of Northwest and Central Switzerland. We retrospectively reviewed the data of all patients with petrochanteric fractures with lesser trochanter displacement. The present study included 20 patients with pertrochanteric femoral fracture and displacement of the lesser trochanter (≥ 20 mm) who underwent surgical treatment at the Kantonsspital Baden between 2013 and 2016 with a follow-up period of more than 6 months. The inclusion criteria were: AO/OTA type 31-A2 and AO/OTA type 31-A3 fractures with lesser trochanter displacement of ≥ 20 mm. Nineteen patients were treated with closed reduction and IM fixation (Stryker Gamma IM Nailing System®, Michigan USA), while one patient was treated with a condylar plate (DePuy Synthes Condyler Plate®, West Chester, Pennsylvania, USA (Table 1).

**Table 1:** Patients demographics

	*n* (%)
No. of patients	20 (100)
Sex	
Male	7 (35)
Female	13 (65)
Age (yrs)	
Range	28–85
Median	74
Body mass index (kg/m^2^)	
Range	17.87–38.86
Median	23.51
Harris hip score	
Range	36.73–100
Median	94.73
WOMAC score	
Range	10–100
Median	93.8
Side of femoral fracture	
Right	11 (55)
Left	9 (45)
Displacement of lesser trochanter (mm)	
Range	20–47
Median	25
Surgical therapy	
Gamma nail	19 (95)
Plate osteosynthesis	1 (5)
Time interval between surgery and examination (days; n = 18)	
Range	196–842
Median	456

### Radiological assessment of fatty infiltration

**Fig. 1:**
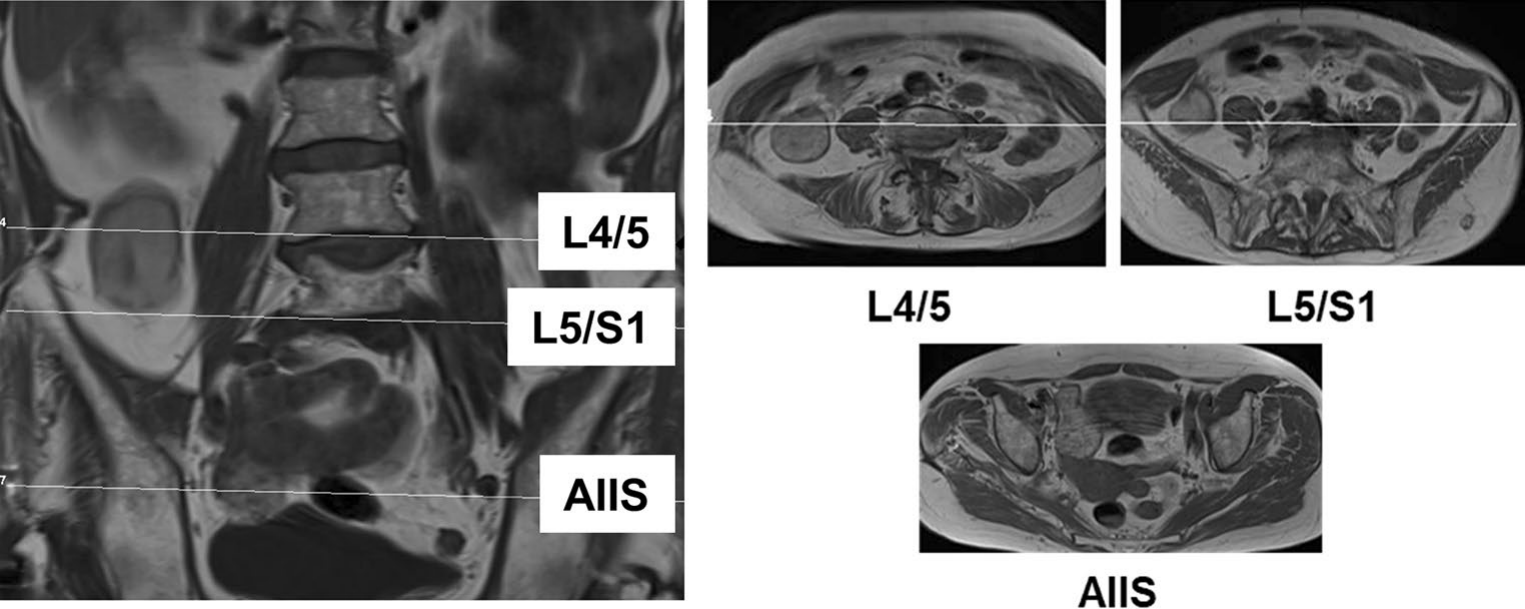
Anatomical landmarks used in MRI-based assessment of fatty infiltration. *MRI* magnetic resonance imaging; *L4/5* lumbar segments 4/5; *L5/S1* lumbosacral segment; *AIIS* anterior inferior iliac spine

More than 6 months postoperatively (median 456 days, range 196–842 days), 18 of the 20 patients (one refused due to claustrophobia, one had a pacemaker which was not suitable for MRI) underwent MR imaging of the hip region for the assessment of muscle quality of the hip flexor muscles (M. iliacus, M. psoas major, M. iliopsoas) as previously described using the four-tiered classification established by Goutallier et al. [12, 13]. All MRI examinations were performed on both the operated and the contralateral side by two experienced radiologists, giving a total of 120 measurements. The fatty infiltration of individual muscles was assessed according to the classification system proposed by Kaniewska et al. (anatomy-based MRI assessment of the iliopsoas muscle complex after petrochanteric femoral fracture, under revision, March 2018). The intervertebral discs at L4/L5 and L5/S1 were used as radiological landmarks for the iliacus and psoas major, respectively, while the anterior inferior iliac spine was used as a landmark for the iliopsoas (Fig. 1). In addition, the mean global fatty degeneration index (GFDI) was calculated for each muscle as previously described for rotator cuff lesions [12, 13].

### Assessment of hip flexor strength

**Fig. 2:**
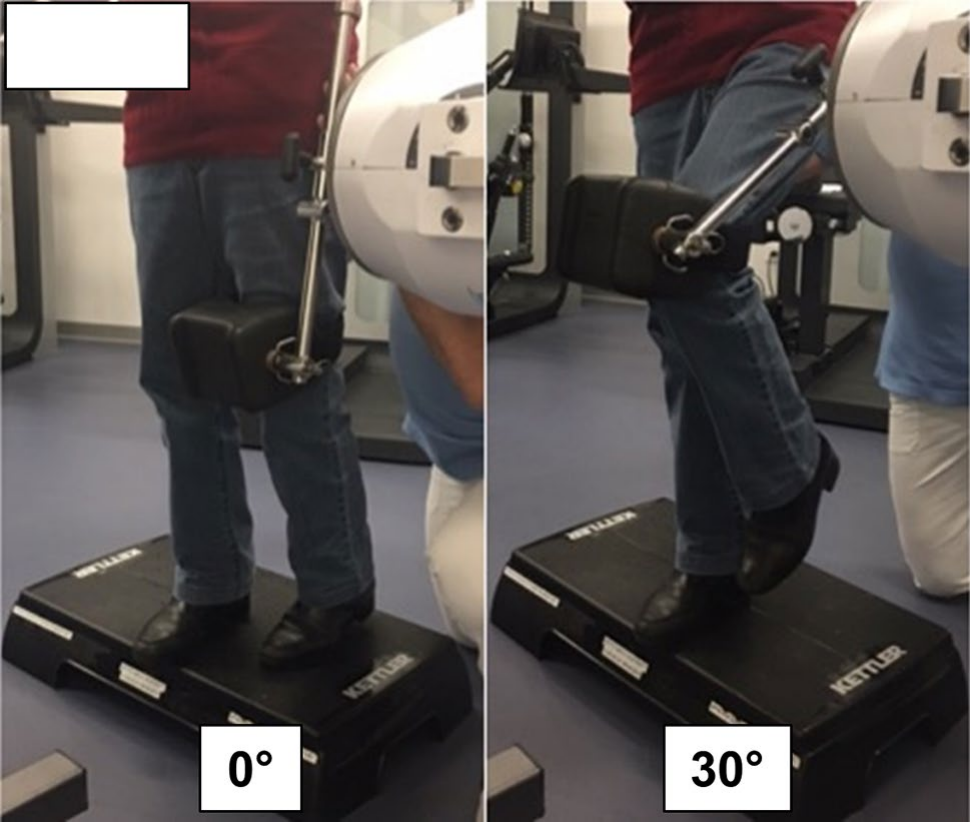
Assessment of hip flexor strength using the Primus RS dynamometer. *Left* image showing the force measurement of the hip flexors at 0° flexion. *Right*: image showing the force measurement of the hip flexors at 30° flexion

At the time of MRI assessment, each patient also underwent clinical examination, evaluation of range of motion (ROM) for both hip joints, assessment of the Harris hip and WOMAC scores, and evaluation of body mass index (BMI); these assessments were also done for the two patients who did not undergo MRI. Hip flexor strength was measured using the Primus RS dynamometer (Fig. 2) on both the operated and the contralateral sides as previously described for at 0° and 30° flexion [14, 15]. The measured values were reported in Newtons (N).

### Statistical analyses

GraphPad Prism software (GraphPad, LaJolla, USA) was used for all statistical analyses. Differences in GFDI, ROM, and hip flexor strength were evaluated using the Student’s *t* test or ANOVA with multiple comparison post-testing. Correlations between GFDI and patient age, ROM, hip flexor strength, and extent of displacement of the lesser trochanter were assessed by logistic regression analyses. For all statistical tests, a p value below 0.05 was considered statistically significant.

## Results

The median age of the patients was 74 years (range 28–84 years). The median time interval between index surgery and MRI assessment was almost 18 months (median 456 days, range 196–842 days). All patients were mobile without walking aids. The median Harris hip score was 94.7 (range 36.7–100), and the median WOMAC score was 93.8 (range 10–100). Conventional radiography showed complete healing of the main trochanteric fragments, with a median displacement of the lesser trochanter of 25 mm (range 20–47 mm).

### Range of motion and hip flexion strength

**Fig. 3:**
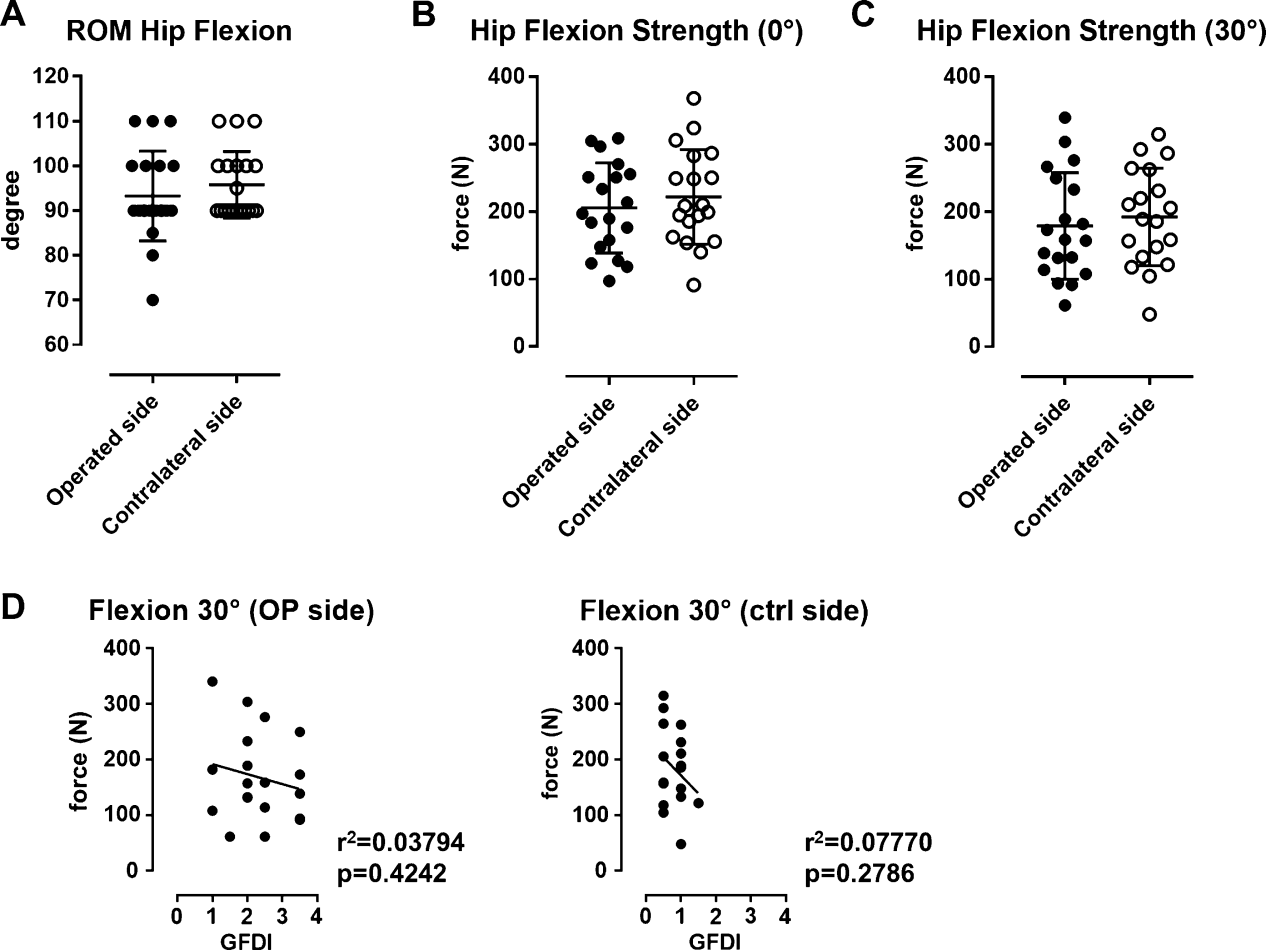
**a**–**c** There were no significant differences in ROM (°) and hip flexion strength (N) at 0° and 30° flexion between the operated vs. the contralateral sides. **d** Linear regression analysis showed no correlation between GFDI and hip flexion strength on both the operated and the contralateral sides. *GFDI* global fatty degeneration index; *ROM* range of motion

There were no significant differences in ROM between the operated and the contralateral hip at 6 months postoperatively (mean 93.25° vs. 95.75°; Fig. 3 A, *p* = 0.3774, Student’s *t* test). Furthermore, the force measurement values were not significantly different regarding hip flexion strength at 0° and at 30° flexion compared with the contralateral side (mean 205.4 N vs. 221.7 N at 0° flexion, and 178.9 N vs. 192.1 N at 30° flexion; *p* = 0.4983 and *p* = 0.5871, respectively, Student’s *t* test) (Fig. 3b, c). There was no significant association between the GFDI and hip flexion strength of the operated side at 0° flexion (*r*^2^ = 0.02346, *p* = 0.5313, linear regression analysis) or 30° flexion (*r*^2^ = 0.03794, *p* = 0.4242, linear regression analysis).

### Global fatty degeneration index (GFDI)

**Fig. 4:**
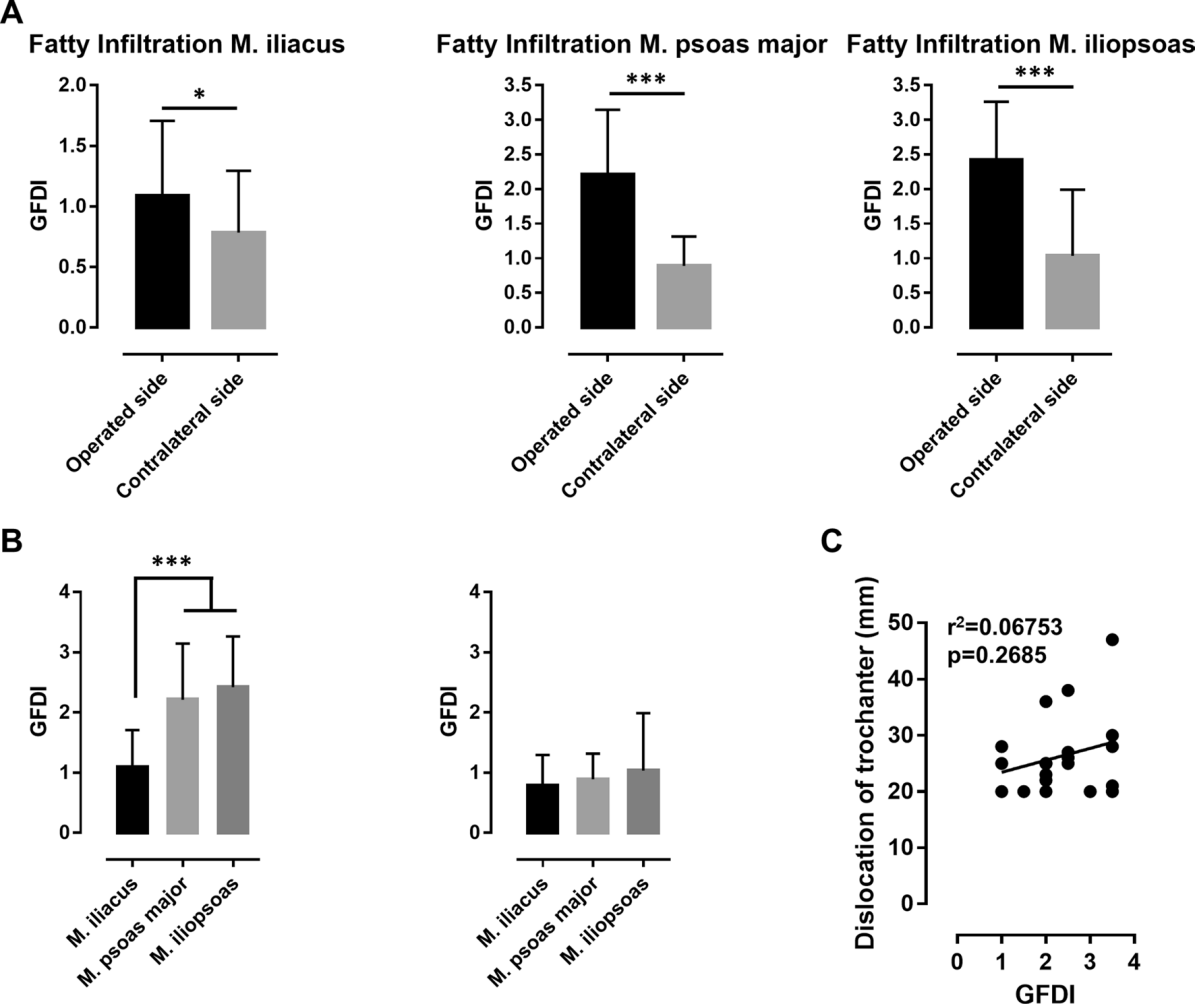
**a** Comparison of the GFDI of the iliacus, psoas major, and iliopsoas muscles on the operated vs. the contralateral sides. **b** Comparison of the GFDI of the iliacus, psoas major, and iliopsoas muscles on each side. **c** Linear regression analysis showed no correlation between GFDI and dislocation of the lesser trochanter. *GFDI* global fatty degeneration index. *p < 0.05; ***p < 0.001

MRI revealed that the GFDI on the operated side was significantly greater than that of the contralateral side for all investigated muscles (Fig. 4a). The GFDI values on the operated sides were 1.085 for the iliacus (contralateral side: 0.7841, *p* = 0.0164, Student’s *t* test), 2.211 for the psoas major (contralateral side: 0.8889, *p* < 0.001, Student’s *t* test), and 2.417 for the iliopsoas (contralateral side: 1.033, *p* < 0.0001, Student’s *t* test). On the operated side, the GFDI values of the iliopsoas and the psoas major were significantly greater than the GFDI of the iliacus (Fig. 4b; *p* < 0.0001, ANOVA with Tukey’s multiple comparisons test).

While the GFD indices on the contralateral side were overall significantly lower, there was a small, but significant higher GFDI in M. iliopsoas compared to M. iliacus (Fig. 4b; *p* < 0.05, ANOVA w/Tukey’s multiple comparisons test). There was no significant correlation between GFDI and the extent of the displacement of the lesser trochanter (Fig. 4 C; *r*^2^ = 0.06753, *p* = 0.2685, linear regression analysis). Interestingly, there was a weak, but significant association between GFDI and patient age (data not shown; *r*^2^ = 0.3874; *p* = 0.0034, linear regression analysis).

## Discussion

The present study reveals that displacement of the lesser trochanter by more than 20 mm leads to significant fatty infiltration of the iliacus and the psoas muscles compared with the non-operated side. Within the musculotendinous unit, the psoas muscle was the most affected. To our knowledge, this is the first description of fatty infiltration of the iliopsoas muscle in a posttraumatic setting. These results are comparable to the findings reported after hip arthroscopy, in which the psoas tendon was tenotomized [16–18]. Blomberg et al. reported that iliacus and psoas atrophy were present in 65 and in 85% of patients, respectively, and that the psoas was more affected than the iliacus [18].

The present study did not confirm the second part of our hypothesis that displacement of the lesser trochanter or fatty infiltration of the iliopsoas would lead to reduced hip flexion force. Even a high grade of fatty infiltration did not negatively affect flexor strength. We could not detect a significant loss in hip flexion force at 0° or 30° flexion. This is surprising, as the iliopsoas is the largest hip flexor. It is notable that the amount of fracture displacement did not affect the severity of fatty infiltration.

The present results correspond to the scarce data in the literature [9, 10, 16, 18, 19]. In 1966, Norcross et al. [10] described that patients are back to normal hip flexion strength within 8 weeks of the operated side compared with the contralateral side. More recently, Aprato et al. [9] compared patients with pertrochanteric fracture with and without displacement of the lesser trochanter and reported that patients with a displaced lesser trochanter showed a significant reduction in hip flexion force in all positions (straight, 90° flexion, and figure-of-four). However, this previous study only included patients younger than 65 years, and the two groups had a different age distribution (mean ages of 52 and 57 years, respectively). In addition, when comparing the operated and non-operated sides (as in the present study), Aprato et al. [9] found a difference in hip flexion force only at 90° of flexion.

Similarly to our observations, a previous study reported that patients with psoas tenotomy after hip arthroscopy show almost normal Harris hip scores, regardless of the amount of muscle atrophy [16]; notably, these patients were significantly younger (mean age 34.7 years) than the patients in the present study with presumably higher functional demands. In contrast, Brandenburg et al. [19] reported a significant reduction in flexion strength of about 20–25% at 90° flexion, but no change in flexor strength in the supine position.

The current literature contains very little data, mostly from case reports [20], on whether it is necessary to perform reduction and fixation of a displaced lesser trochanter fragment in intertrochanteric fractures. Irrespective of the theoretical loss of flexion strength, reduction of the lesser trochanter could be advocated to increase mechanical support. This would reduce the risk of loss of fracture reduction, penetration of the hip screw or varus deformity. In fact, there is biomechanical evidence from an in vitro study [21] that refixation of the lesser trochanter increases primary stability of pertrochanteric fracture osteosynthesis. In a recent published retrospective study, 22 patients with intertrochanteric fractures and lesser trochanter fragment displacement where treated surgically with proximal IM nailing and with a wiring technique for reduction and fixation of the lesser trochanter [22]. They found good clinical outcomes in 18 patients achieving hip function prior to the trauma, whereas four patients remained with a functional deficit. The authors postulate that increased posteromedial stability in these unstable fractures results in a reliable bone union in all patients and allows early ambulation. However, this study lacks a control group without fixation of the lesser trochanter to draw such conclusions.

In conclusion, the present study revealed, that a displaced fracture of the lesser trochanter leads to significant fatty infiltration of the iliopsoas musculotendinous unit, and that this atrophy does not significantly reduce hip flexion force. The loss of this large muscle may be compensated by better recruitment or hypertrophy of other hip flexor muscles. Therefore, current practice guidelines recommend not addressing the displaced lesser trochanter. Our findings support this practice, at least in elderly patients with low functional demands.

### Limitations

Our study has limitations. First, the number of enrolled patients was a relatively low. This may explain, why we were not able to find differences of flexion force between the fractured and unaffected side. However, the number was sufficient to find significant fatty infiltration of the most important hip flexor on the fractured hip. Second, the flexion strength was only measured in the upright position and at 30° of hip flexion. Measurements in a higher flexion position (e.g., at 90° flexion) were not made. However, the clinical relevance of measurements in such a flexed position is questionable, as walking on flat ground or walking upstairs requires only a limited range of flexion in the hip joint.

*Future directions* may look at the timely sequence of the development of fatty infiltration and whether other muscles compensate the deficit through reactive hypertrophy.
